# Hypoxia induced lactate acidosis modulates tumor microenvironment and lipid reprogramming to sustain the cancer cell survival

**DOI:** 10.3389/fonc.2023.1034205

**Published:** 2023-01-25

**Authors:** Lakhveer Singh, Lakshmi Nair, Dinesh Kumar, Mandeep Kumar Arora, Sakshi Bajaj, Manoj Gadewar, Shashank Shekher Mishra, Santosh Kumar Rath, Amit Kumar Dubey, Gaurav Kaithwas, Manjusha Choudhary, Manjari Singh

**Affiliations:** ^1^ School of Pharmaceutical & Population Health Informatics, DIT University, Dehradun, India; ^2^ Department of Pharmaceutical Science, Assam University (A Central University), Silchar, Assam, India; ^3^ Department of Pharmaceutical Sciences, Central University of Haryana, Mahendergarh, Haryana, India; ^4^ Chaudhary Devi Lal College of Pharmacy, Yamuna Nagar, India; ^5^ School of Medical and Allied Sciences, KR Mangalam University, Gurgaon, India; ^6^ Department of Pharmaceutical Science, Babasaheb Bhimrao Ambedkar University, Lucknow, India; ^7^ University Institute of Pharmaceutical Sciences, Kurukshetra University, Kurukshetra, India

**Keywords:** hypoxia, HIF-1α, lactate, angiogenesis, invasiveness, resistance, Immunity, Lipid reprogramming

## Abstract

It is well known that solid hypoxic tumour cells oxidise glucose through glycolysis, and the end product of this pathway is fermented into lactate which accumulates in the tumour microenvironment (TME). Initially, it was proclaimed that cancer cells cannot use lactate; therefore, they dump it into the TME and subsequently augment the acidity of the tumour milieu. Furthermore, the TME acts as a lactate sink with stope variable amount of lactate in different pathophysiological condition. Regardless of the amount of lactate pumped out within TME, it disappears immediately which still remains an unresolved puzzle. Recent findings have paved pathway in exploring the main role of lactate acidosis in TME. Cancer cells utilise lactate in the *de novo* fatty acid synthesis pathway to initiate angiogenesis and invasiveness, and lactate also plays a crucial role in the suppression of immunity. Furthermore, lactate re-programme the lipid biosynthetic pathway to develop a metabolic symbiosis in normoxic, moderately hypoxic and severely hypoxic cancer cells. For instance: severely hypoxic cancer cells enable to synthesizing poly unsaturated fatty acids (PUFA) in oxygen scarcity secretes excess of lactate in TME. Lactate from TME is taken up by the normoxic cancer cells whereas it is converted back to PUFAs after a sequence of reactions and then liberated in the TME to be utilized in the severely hypoxic cancer cells. Although much is known about the role of lactate in these biological processes, the exact molecular pathways that are involved remain unclear. This review attempts to understand the molecular pathways exploited by lactate to initiate angiogenesis, invasiveness, suppression of immunity and cause re-programming of lipid synthesis. This review will help the researchers to develop proper understanding of lactate associated bimodal regulations of TME.

## 1 Introduction

Chemotherapy of solid malignant tumours has become a major challenge because of the development of resistance due to hypoxia-a condition characterised by lower amount of oxygen. In addition to imparting resistance to chemotherapy, it helps tumour cells acquire the most favourable environment, which supports their survival, even in oxygen- and nutrient-deficient environments (1). Various studies have reported that hypoxia-activated hypoxia-induced factor-1α (HIF-1α) functions at the gene level to enhance angiogenesis, metastasis, and invasiveness of cancer cells (2). HIF-1α also alters glucose, fatty acid, and amino acid metabolism to support cancer cell survival (3). Reprogramming of glycolysis has already been reported by Warburg group. They stated that cancer cells metabolise glucose only through glycolysis, even if there is sufficient oxygen supply which results in excessive accumulation of lactate (4). The continuous release of lactate in the tumour microenvironment (TME) makes it even more acidic (5). For many years, lactate has been recognised as a metabolic waste product which is toxic to cancer cells and is pumped out in the TME, but how TME pumped out the dump lactate is still not clear. Later, it was reported that malignant cells can utilise lactate secreted by nearby cancer cells. Recently, Brandon et al. reported that lactate fuels the tricarboxylic acid (TCA) cycle in normoxic cancer cells. *In-Vitro* and *In Vivo* studies on non-small cell lung carcinoma cells (NSC-LCs) demonstrated that, similar to glucose metabolism, lactate can also be utilised for energy production if the cancer cells have an adequate oxygen supply. Previous studies have already reported that tissue architecture and anatomical location also influence the use of lactate as a fuel. For instance, lung carcinoma cells can oxidise lactate in the TCA because lung tissue has a high level of perfusion and oxygenation (6). Later, Hui et al. in their study reported that glucose-derived lactate indirectly feeds into the TCA cycle. They carefully examined the fluxes of C^13^-labelled lactate in mice *via* intravenous infusion. The results of this study showed the highest circulatory lactate turnover flux. Moreover, the circulatory turnover flux of lactate exceeded that of glucose by 2.5-fold in fasted mice and 1.1 fold in fed mice. To determine whether cancer cells can use lactate, they injected C^13^-labelled lactate, glutamate, and alanine into genetically engineered lung and pancreatic tumour cells, and noted that the circulating lactate input in the citric acid cycle outpaced that of glucose. These studies clearly demonstrate that cancer cells can utilise circulating lactate (7). However, the mechanism through which cancer cells utilise lactate remains unclear.

Numerous studies have demonstrated an immunosuppressive role of lactate in cancer. It has been previously reported that a lactate-derived acidic TME in cancer has the potential to abolish cytotoxic T cells (CD8+ T cells) and natural killer (NK) cell anticancer immune responses. The acidic TME also interferes with the antigen presentation process of dendritic cells (DC) and halts maturation and differentiation (8). However, it remains a matter of discussion that how lactate protect cancer cells from the innate and adaptive immune responses and is there any way to trigger anticancer immune cells by regulating lactate acidosis?

Additionally, lactate is involved in angiogenesis and metastasis. Zhou et al. reported that lactate promotes neovascularization and neurogenesis *via* the Nuclear factor kappa-B (NF-kB) signalling lane. Although several studies have established the link between lactate and angiogenesis but induction of angiogenesis by lactate mediated signalling through activation of NF-kB pathway has not been well understood (9).

Lactate also help the cancer cell’s invasion into the adjacent organs. Previous studies have delineated how lactate initiates invasiveness by inducing claudin-1 (Cln-1) expression through mitochondrial respiratory defects. However, the exact underlying mechanisms remain unknown (10). Later, An et al. also reported that elevated cytosolic enzymes, such as lactate dehydrogenase (LDHA), help tumour cells to become invasive. The results of this study confirmed that the overexpression of LDHA in pituitary oedema promotes cell invasion and proliferation (11).

Previously, in our lab, we have reported that hypoxia upregulates the fatty acid synthesis in mammary gland cancer cells. Results of the immunoblotting and metabolomics studies documented increased level of HIF-1α, sterol regulatory element binding proteins (SREBP) and fatty acid synthase (FASN) while level of prolyl hydroxylase-2 (PHD-2) was reduced significantly (12–14). Serum metabolomics profile also showed increased level of lactate and low density lipoproteins/very low density lipoproteins (LDL/LDL) and poly unsaturated fatty acids. We performed few more studies in the same direction and every time we noted increased level of lactate and fatty acids in carcinogen treated animals (15). Further we reported that hypoxia and lipid biosynthesis can be curtailed through activation of PHD-2. Interestingly we observed reduced level of HIF-1α, lactate, SREBP and fatty acids upon chemical activation of PHD-2 (16, 17). Based upon our observation we developed another hypothesis that lactate can be incorporated into lipid biosynthetic pathways because glucose alone is inefficient in meeting the increasing demand for fatty acids in malignant cells. But which pathway is exploited by cancer cells for incorporation of lactate into fatty acid synthesis-is still unknown.

From the above discussion, it is evident that lactate is the major metabolite that helps malignant cells suppress immunity, impart resistance to chemotherapy, and promote angiogenesis and metastasis. Although much has been discussed regarding the role of lactate in the abovementioned process, there are still several gaps in the literature that raise various questions. The current review aims to fill the gap in understanding the role of lactate in the aggressive transformation of malignant solid tumours and to answer the unrevealed questions. This review focuses on the synthesis of lactate-derived fatty acids in detail and its association as well as significant role in TME.

## 2 Development of hypoxia and activation of HIF-1α in solid tumours

Cancer cells immediately develop hypoxia as their distance continues to increase owing to an increase in tumour size. Owing to continuous pushing away from blood vessels, cancer cells face nutrient and oxygen deficiencies called hypoxia (18, 3). Normal cells undergo apoptosis in nutrient-deficient environments; however, cancer cells are immortal. They take help from HIF-1α (a cytoplasmic protein expressed ubiquitously) which is activated in an oxygen-deficient environment, and is transported into the nucleus after dimerisation with their cytoplasmic subunits (19). In the nucleus, dimerised HIF-1α regulates the expression of several genes that play unique roles in cell cycle regulation, apoptosis, angiogenesis, metastasis, and invasiveness (20). HIFs-α also monitor the metabolism of glucose, fatty acid and amino acids specifically and wisely to sustain the survival of normoxic and hypoxic cancer cells in the TME. All of the aforementioned effects are discussed in detail in the preceding section of this review.

## 3 HIF-1α shifts the metabolism of glucose through glycolysis to enhance production of lactate

Firstly, Otto Warburg reported that cancer cells metabolise glucose under aerobic conditions. The incomplete oxidation of glucose to pyruvate leads to the accumulation of lactic acid which is pumped into the TME. Continuous pumping of lactate to the extracellular compartments increases the acidity of the TME, which indirectly functions under the instructions of HIF-1α to reprogram fatty acid and amino acid metabolism as a signalling molecule to induce metastasis, angiogenesis, invasiveness, and suppression of immunity (21). The exact molecular pathways exploited by the lactate to induce angiogenesis, invasiveness, immune suppression and fatty acid synthesis is discussed in much detail in the preceding section under individual headings.

## 4 Role of lactate in angiogenesis

An increase in the size of the tumour not only increases the suffering of patients but it also increases the proportion of hypoxic cancer cells. With an increase in tumour size, cells that were initially located near to blood vessels were slowly displaced away from the blood vessels. These cancer cells have a limited supply of oxygen and nutrients and become hypoxic (22). Usually, normal cells die under oxygen and nutrient deficiency conditions, but cancer cells uses alternative machinery; hence, new blood vessels are formed, and the process is known as angiogenesis (23). Several studies have demonstrated that lactate promotes angiogenesis but how does lactate initiates angiogenesis in cancer cells is yet unknown. Previous studies have reported that HIF-1α regulates the activation of various pro-angiogenic factors, such as vascular endothelial growth factor (VEGF), plasminogen activator inhibitor-1, platelet-derived growth factor-B (PDGF-B), and overexpression of VEGF receptor genes, especially Fms-related receptor tyrosine kinases (FLT-1 and FLK-1), matrix metalloproteinases (MMP-2), TIE2(a receptor tyrosine kinase) receptor, and angiopoietins (ANG-1 and ANG-2). It has been shown that 47 of the total pro-angiogenic factors reported to date are regulated by HIF-1α (24, 25). Kishimoto et al. reported that neovascularization is indispensable for tumour growth and development. *In Vivo* and *In Vitro* studies on melanoma cells have shown that the angiogenic factor ANG is upregulated many-fold in hypoxic environments (26). Another study by Wang et al. reported that hypoxia effectively supports the initiation of angiogenesis in cancer cells. They cultured mouse breast carcinoma 4T1 cells under normoxic (21% oxygen) and hypoxic (1% oxygen) conditions. The results of the mRNA expression showed a higher level of angiogenesis-associated factors, such as VEGF, fibroblast growth factor-2 (FGF-2), PDGF-B, placental growth factor (PIGF), and ANG-2, in 4T1 cells cultivated in an oxygen-deficient environment. Hypoxic 4T1 cells treated with metformin showed reduced expression of these angiogenic factors and reduced the hypoxic prove-positive area. Hypoxia prove positive area is a tumor region highlighted by the antibodies. The results of this study are well supported by *In Vivo* experiments. Breast carcinoma was induced by injecting 4T1 cells into mammary gland tissue. Immunohistochemistry analysis demonstrated enhanced expression of proangiogenic factors which got significantly reduced after metformin treatment (27, 28). These studies clearly demonstrate the role of hypoxia in angiogenesis but how hypoxia regulates angiogenesis still not clear. More recently, it was reported that lactate in the tumour microenvironment signals neovascularization in hypoxic cancer cells. Consequently, decreased vascular perfusion in malignant tumours resulted in lower oxygen and glucose levels (29). To survive in this harsh environment, malignant tumour cells oxidise glucose anaerobically which results in excess production of lactate and a reduction in pH in the tumour microenvironment (30). The acidic tumour microenvironment stimulates cancer cells to secrete a wide variety of angiogenic factors to re-establish local blood supply. The study also reported that tumor associated macrophages (TAM) secrete similar pre-angiogenic factors under stressful conditions (31). Sonveaux et al. cultured bovine aortic endothelial cells (BAECs) to delineate the influence of lactate on angiogenesis in hypoxic cancer cells. NMR metabolomics revealed increased lactate levels in the hypoxic cells. Immunohistochemistry and western blotting revealed increased expression of monocarboxylate transporter-I (MCT-1 and 4). Hypoxic cancer cells oxidise glucose anaerobically, and the resultant lactate is pumped into the TME through MCT-4. Lactate is transported from hypoxic cancer cells to normoxic cancer cells, where it is absorbed by oxidative cancer cells (through the MCT-1 transporter) and utilised in the citric acid cycle for energy production. Furthermore, they performed *In Vivo* experiment to confirm whether lactate has any role in angiogenesis; they implanted Matrigel plugs subcutaneously in the flanks of mice. CD31 immunolabelling was performed and observed a 10 fold increase in endothelial colonisation (32). Kes et al. filled this gap and described the role of lactate in angiogenesis through a comprehensive review. They reported that lactate released by hypoxic malignant cells is taken up by tumour-associated macrophages (TAM) that secrete various cytokines to initiate neovascularization in cancer cells (33, 34). Although the above studies clearly demonstrate that lactate directly communicates with endothelial cells to initiate angiogenesis, the exact signalling pathways involved have not been properly resolved. Guo-Xiang and Kazlauskas cultured human umbilical endothelial cells (HUVECs) of veins to explore the signalling pathway using lactate to induce angiogenesis. The results of qRT-PCR and western blotting showed that lactate enhanced the expression of Axl, Tie2, and VEGFR-2 in endothelial cells, and these receptors were further stimulated by an autocrine mechanism. The expression of Gas6, Ang1, and VEGF increased several-fold in lactate-treated cells which further acted on Axl, Tie2, and VEGFR-2 receptors. Activation of Axl, Tie2, and VEGFR-2 receptors further causes phosphorylation of PI3K and Akt, thus initiating angiogenesis (35). Axl, Tie2, and VEGFR-2 belong to a family of tyrosine kinase receptors and are involved in the regulation of diverse activities in endothelial cells. Tie1 and Tie2, endothelial cell-specific tyrosine kinase receptors, are indispensable for the maturation and remodelling of lymphatic and blood vessels (36). ANG-1 and ANG-2 are important ligands for the Tie2 receptors (37). Asahara et al. performed a corneal micropocket assay to explore the role of angiopoietin-1 and 2 in neovascularization. The results of the study reported that neither angiopoietin 1 nor 2 alone promoted angiogenesis, whereas the addition of VEGF alone initiated angiogenesis. It was also observed that the addition of angiopoietin-1 and 2 along with VEGF more aggressively initiated the process of angiogenesis (38). These studies not only revealed the role of ANGs but also demonstrated that VEGF is equally important for initiating angiogenesis (39).

Axl is another important tyrosine kinase receptor present in approximately all body tissues (40). Previous studies have reported that Axl plays a pivotal role in metastasis and angiogenesis in various cancers (41). It has also been reported that Gas6/Axl signalling pathways inhibit metastasis, invasion, angiogenesis, immune regulation, and stem cell maintenance (42). Axl overexpression has been reported in patients with NSCLC, along with poor invasion, metastasis, and drug resistance (43).

From these findings, it can be concluded that energy stress in hypoxic cancer cells initiates angiogenesis. Hypoxia-activated HIF-1α helps hypoxic cancer cells to overcome energy stress by inducing angiogenesis. HIF-1α can initiate neovascularisation in hypoxic cancer cells by enhancing the gene expression of VEGF by or by utilizing lactate as a signalling molecule (44, 19).

**Figure 1:**
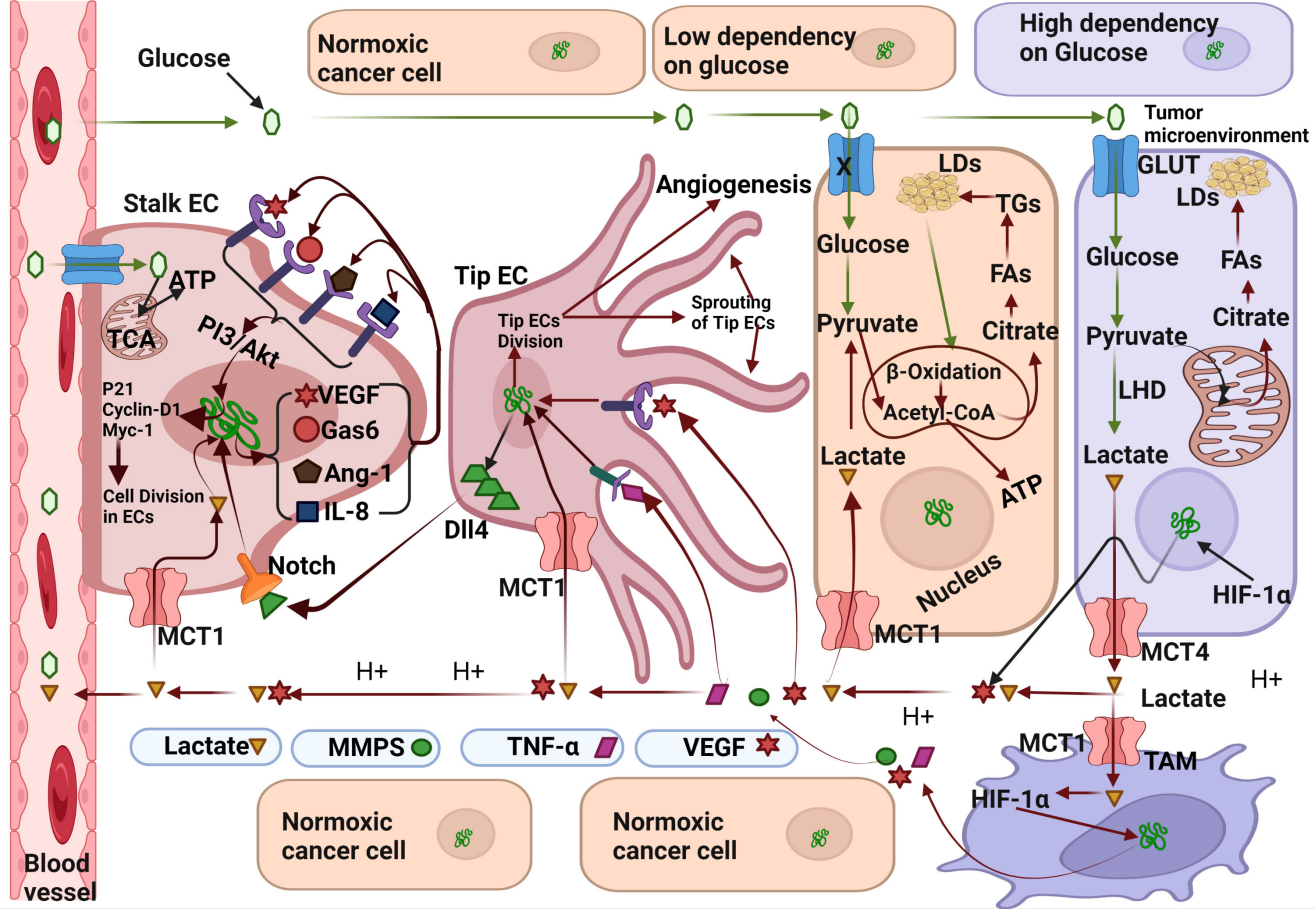
Molecular mechanism exploited by Lactate for angiogenesis induction. Hypoxic cancer cells can metabolise glucose only by glycolysis resulting in accumulation of lactate which is secreted in the tumor microenvironment (TME) through the monocarboxylate transporter-1(MCT-4). Lactate from TME flows towards nearby blood vessels and enters endothelial cells (EC) through MCT-1 transporter. After entering stalk epithelial cells, lactate upregulates the expression various pro-angiogenic genes like-Gas6, VEGF, Ang-1, and IL-8 which acts in the autocrine mechanism on their respective receptors. Gas-6 binds to the Tie2 receptor, VEGF binds to the VEFR2 receptor, Ang-1 binds to the Axl receptor, and IL-8 binds to the IL-8 receptor. Activation of VEGFR2, Tie2, IL-8 and Ang-1 receptors initiate cell division in stalk epithelial cells by activation of Cyclin-D/Myc-1/p21 genes and subsequently lead to the differentiation of stalk epithelial cells into new blood vessels. Fatty acids synthesised from lactate derived citrate in normoxic cancer cells transported towards the stalk ECs wherein used in β-Oxidation to full fill their energy needs. Lactate also enters tip epithelial cells and activates various genes which also regulate the cells division of tip ECs. A fraction of lactate also enters the tumor associated macrophages (TAM) through MCT-1 transporter and activates the inflammatory mediators like TNF-α, Vascular Endothelial Growth Factor and Metalloproteinases (MMPs). MMPs digest the stromal proteins, clears the road to sprouting blood vessels. Binding of VEGF and TNF-α also upregulates the expression of Delta-like-ligand 4(Dll4) which further regulate the differentiation of adjacent stalk ECs *via* NOTCH signalling pathway. Created by Biorender.

A.VEGF andDll4 mediated angiogenesis: In this mechanism the activation of various genes, such as VEGF which when expressed by hypoxic cancer cells, is secreted into the TME. VEGR from TME binds to VEGFR1/2) present on the endothelial tip cells and triggers the release of Delta-like ligand-4 (Dll4) present on the endothelial tip cells (**Figure 1**) (45). The Dll4 ligand binds to the Notch receptor present on stalk endothelial cells and triggers their proliferation and the subsequent sprouting of blood vessels (46). Although previous studies have reported that Dll4 acts a negative regulator of angiogenesis, exact role of Dll4 in angiogenesis remains undetermined.

B.Lactate mediated angiogenesis: Lactate can induce angiogenesis by both mechanism-directly and indirectly

B.1.Indirect mechanism: Lactate can indirectly induce angiogenesis by acting as a signalling molecule. Excess lactate produced by hypoxic cancer cells flows back into blood vessels and is taken up by the normoxic cancer cells. It is reused in oxidative phosphorylation for energy production, and some portion enters TAM and endothelial cells (EC) (47). Lactate can enter TAM directly through the MCT-1 transporter or act on the G-protein coupled receptor(GPCR) as a signalling molecule (48). Following the activation of GPCR and subsequent activation of adenyl cyclase, cAMP and Inducible cAMP Early Repressor (ICER) lead to the activation of various genes involved in the anti-inflammatory process initiated by TAM like Arginase-1(Arg-1), resistin-like molecule alpha1(Fizz-1),CD206, and VEGF molecules. Once inside the TAM, lactate acts through various mechanisms to an anti-inflammatory mechanism which moves towards the nearby blood vessel and binds to their defined receptors present on endothelial cells, subsequently triggers the sprouting of stalk/tip EC cells into new blood vessels (33).

B.2.Direct mechanism: Another mechanism that initiates angiogenesis involves the direct entry of lactate into endothelial cells through the MCT-1 transporter followed by the activation of various genes like Gas6,VEGF,Ang-1 and IL-8 (49). The gene products binds on their respective receptors (VEGFR, Tie2 and Axl) in autocrine mechanism through PI3/Akt pathways and regulate the expression of various genes involved in cell cycle of ECs like cycline-D1, P21 and Myc-1. Activation of cyclin-D1 triggers the DNA replication and thus mitosis in ECs. Continuous division in ECs eventually develop new blood vessels. This is how lactate initiate angiogenesis in hypoxic tumors. Inhibition of lactate circulation in TME can prevent angiogenesis in malignant tumors.

## 5 Role of lactate in induction of invasiveness

Invasiveness is another characteristic feature of cancer cells by which they invade nearby tissues after reaching a reasonable size. A continuous increase in tumour size eventually leads to a breach in the barriers between adjacent tissue cells (50). Invasiveness is also the first indication for the development of secondary tumours and distant metastases (51).

Basement remodelling and EMT are two well-known hallmarks of invasive behaviour in tumour cells. A considerable number of studies have documented the role of various factors in EMT; however, the role of lactate in tumour invasiveness is not well understood (52). Before discussing the role of lactate in invasiveness, we should take a look on the micro architecture of the basement membrane and epithelial layers.

The basement membrane is a layer of connective tissue just below the epithelial layers, comprising of collagen IV, collagen VII, and glycoproteins which provide mechanical support to tissue cells. The microscopic view of the epithelial layer shows that all cells that have epithelial tissue adhere to each other which is supported by various types of micronised junction proteins, such as adherent junctions, desmosomes, and junctions. Desmosomes remain connected to each other through intermediate filaments of cytokeratin, whereas cortical bundles allow the adherent junction to be in position. Integrin proteins linked to cytokeratin inside the cytoplasm help epithelial cells adhere to the basement membrane (53). During EMT, epithelial cells lose integrin proteins and detach themselves from the basement membrane.

However, the regulation and initiation of cancer cell invasiveness remain unclear. Recent studies have made remarkable progress in delineating the roles of various metabolic intermediates, among which lactate is the major metabolite involved in the induction of invasiveness in cancer cells. June-Hyungkim, while working on hepatoma cells, reported that lactate dehydrogenase B (LDHB) plays a compelling role in the process of invasiveness of hepatoma cells by activating the tight junction protein claudin-1 (Cln-1). This was the first study which documented the role of lactate in invasiveness (54).

Corbet et al., with their studies over cell line as well as on human patients suffering from metastatic cancer demonstrated that lactate acidosis activates the expression of transforming growth factor-β (TGF-β) which acts on the TGF-β receptor and subsequently contributes to the process of EMT of cancer *via* two pathways. First, TGF-β signals the activation of pSmaid2/3 which after being acetylated activates Snail to enhance activation of gene zinc finger E-box-binding homeobox 1 (ZEB1) activation. ZEB1 activation upregulates TGF-β expression. ZEB1 activates the Cadherin-2 (CDH2) and vimentin (VIM) genes, contributing to the development of anoikic resistance and invasiveness. Second, TGF-β also translocate CD36-a lipid transporter which transports long-chain fatty acids (LCFA) from extracellular sources. Accumulated LCFA perform several functions in the malignant cells. A portion of the LCFA is converted into Triacylglycerol’s by the combination of diacylglycerols and Acetyl-CoA, which is stored as lipid droplets (LDs) in the cytosol. LDs also contribute to anoikic resistance. Some portions of LCFA is broken down into acetyl-CoA and transported to the mitochondria for use in β-oxidation for ATP production. Excess acetyl-CoA released from fatty acids (released from LDs) is further carry out the acetylation of Smad2/3 which activates ZEB1 and CDH2/VIM. Therefore, this study also gives a knowledge that why cancer cells require larger quantities of fatty acids than normal cells (55).

Kexin Sun et al, delineated the role of lactate and TGF-β in EMT process and cancer progression. This study was conducted on cancer-associated fibroblasts (CAF) and the human breast cancer cell line MDA-MB-231, as well as on nude mice, to unveil the role of oxidised ataxia-telangiectasia (ATM) in the regulation of glycolysis during hypoxia. Western blotting and immunohistochemistry revealed that CAF grown under hypoxic conditions showed higher expression of Glucose Transporter-1 (GLUT-1). Interestingly, higher expression of Oxidised ATM in a double-strand break (DSB)-independent manner was observed in CAFs under hypoxic conditions. Furthermore, to benefit cancer cells, oxidised ATM in CAF phosphorylates the Serine490 amino acid of GLUT-1 and thus promotes its translocation to the plasma membrane. This enhanced glucose uptake and utilisation in CAF results in the excess secretion of lactate in the tumour microenvironment. Co-culture of MDA-MB-231 and BT549 cells with CAF showed high levels of TGF-β, phosphorylated P38, MMP2, and MMP9. From this, the author inferred that excess glucose metabolism in CAF resulted in lactate accumulation which is pumped in the TME, where it acts as a coupling metabolite and acts as a signalling molecule to enhance the expression of TGF-β1 and phosphorylated P38, MMP2, and MMP9 in order to accelerate the process of invasiveness (56).

Rattigan and colleague reported in their study that tumour cells and cancer associated fibroblasts and stromal cells cooperate each other in the TME leading to mutual existence. Glycolytic cells in the TME metabolise glucose through glycolysis and excess of lactate generated by these cells is readily taken up by the CAFs which is further converted to citrate (57). Konstantin et al. in their study reported that citrate, supplied by cancer-associated stromal cells, is indispensable for cancer cell metastasis. It has been reported that CAF express more citrate carriers on their surfaces which is purposely used for more citrate uptake to be incorporated for fatty acid synthesis or to fuel the citric acid cycle (58). Whitaker-Menezes et al. previously reported that tumour cells and associated fibroblasts can develop metabolic symbiosis to meet their energy requirements (59). Therefore, from the above studies, we can speculate that CAF might use lactate from hypoxic cancer cells and directly convert it into citrate which is further secreted in the TME and readily absorbed by nearby tumour cells for lipid synthesis (60).

In another study conducted by Young-Kyoung et al., on hepatoma cells (SNU354 and SNU423) reported that extracellular lactate can induced invasiveness. The study revealed that glycolytic tumour cells secrete excessive lactate, which enters nearby OXOPHOS cells, interferes with mitochondrial ribosomal proteins, and reduces the expression of mitochondrial ribosomal proteins L13 (MRPL13), leading to defective OXOPHOS and ROS generation. Excessive ROS activated nuclear Cln-1 gene expression and formation of Cln-1 protein which ultimately takes part in invasiveness (61). The study further validated the role of lactate in EMT process.

Lactate also causes the polarisation and activation of macrophages in solid tumours, such as pituitary adenomas, to initiate invasion and infiltration. Lactate from the TME activates macrophages through the mTORC2/ERK pathway and activated macrophages release CCL17 which initiates EMT through the CCL17/CCL4/mTORC1 pathway (62). A previous study conducted by Lin et al. also reported that lactate can induce EMT in cancer cells by activating TAM. TAM in TME secrete CCL5 which induces angiogenesis and EMT in cancer cells. A previous study also established a relationship between TGF-β and CCL5 and proved that TGF1β uses CCL5 to enhance glycolysis in cancer cells (63).

**Figure 2:**
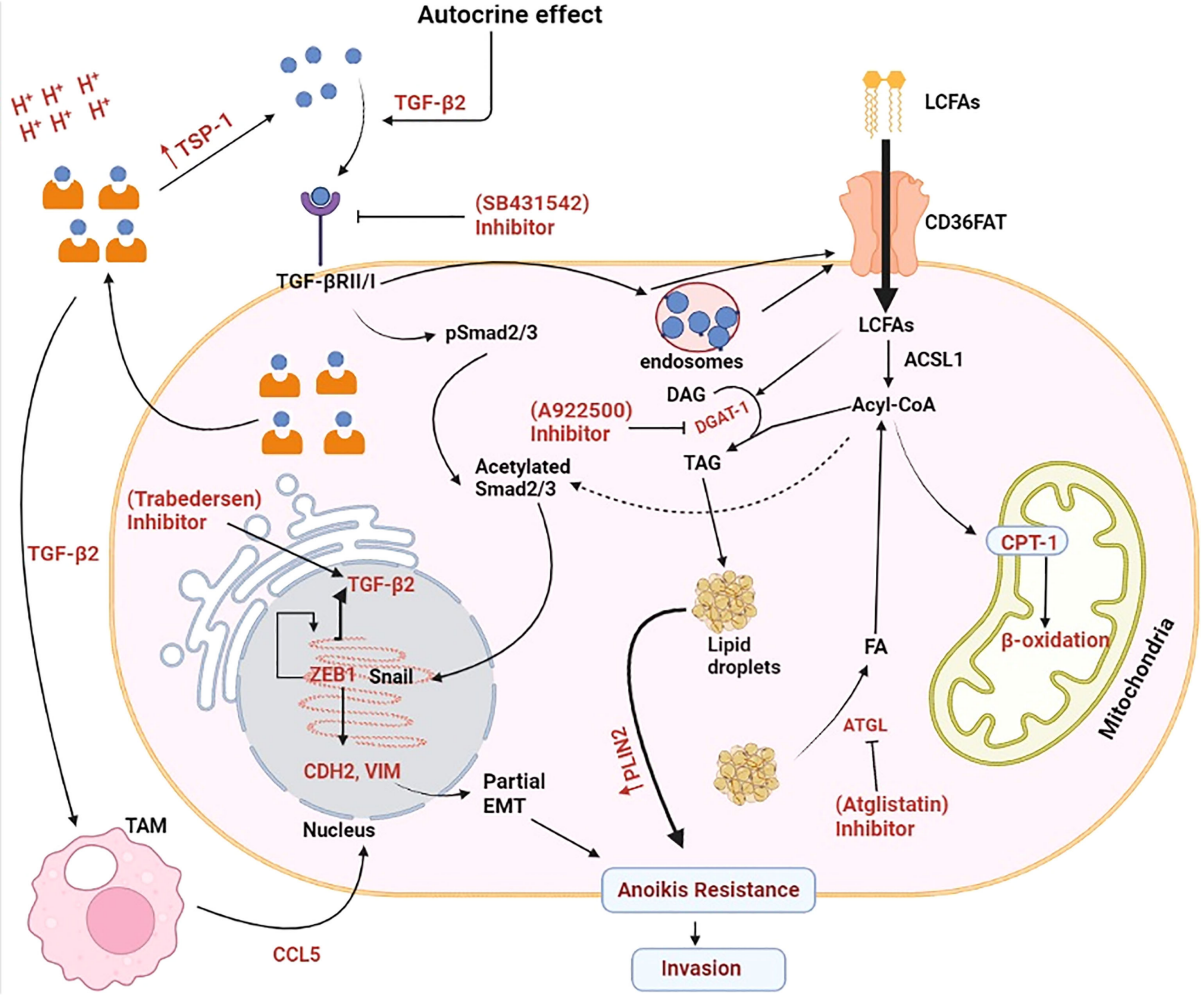
Molecular mechanism exploited by lactate for enhancing invasiveness in cancer cells. Lactate-induced acidosis augments epithelial-to-mesenchymal transition in solid tumours. Lactate in the tumor microenvironment (TME) enhances the synthesis and secretion of tumour growth factor-β2 (TGF-β2) which acts on tumour cells *via* an autocrine mechanism and phosphorylates pSmad2/3. After acetylation, pSmad2/3 activates Snail which enters the nucleus and enhances gene expression of N-Cadherin’s (CDH2) and Vimentin (VIM). CDH2 protein enhances tumor cell motility and migration. VIM belongs to type-IIIrd intermediate filament proteins that maintains cell integrity and plays important role in cell migration, motility and adhesion and subsequently metastasis. TGF-β2 mediated overexpression of CAD and VIM proteins induces partial epithelial to mesenchymal transition (EMT) and Anoikis resistance in cancer cells and finally its transformation into invasive carcinoma cells. Tumour-associated macrophages (TAM) also assist in the EMT process by secreting CCL-5(chemokine cytokine) which further enhances the synthesis and secretion of TGF- β2. Long chain fatty acid (LCFA) taken from external sources provide Acyl-CoA responsible for the acetylation of pSmad2/3. Excess long chain fatty acids (LCFA) is stored as a lipid droplets (LDs) which ensures continuous supply of Acyl-CoA required for acetylation of Smad2/3 proteins. Increasing demand of energy in the form of ATP during EMT process is fulfilled by the β-oxidation of fatty acids released from the stored LDs. ACSL1, Acyl-CoA synthetase; ATGL, Adipose triglyceride lipase; CPT1, Carnitine palmitoyltransferase 1; DAG, Diacylglycerol; FA, Fatty acids; PLIN2, Perilipin 2; TAG:,Triacylglycerol; TSP-1, Thrombospondin-1. Created by Biorender.

This can be concluded from the above discussion that lactate acidosis can initiate invasiveness through activation of TGF-β. Lactate acidosis in TME induces the upregulation of TGF-β in hypoxic cancer cells which is transported outside in the TME. TGF-β from TME acts on TGF-β receptors present on same cancer cells in autocrine mechanism. TGF-β receptor signalling leads phosphorylation of Smad2/3 and subsequently its acetylation. Acetylated Smad2/3 enters the nucleus where it regulates the expression of CDH2 and VIM genes. Protein product of these genes further participate in the EMT process. Acetyl-CoA required for acetylation of Smad2/3 is provided by the long chain fatty acids taken from dietary sources. Triglycerides released from the lipid droplets (LDs) can also confer the acetyl-CoA. High energy demand during EMT process is fulfilled by the β-oxidation of fatty acids released from the LDs (**Figure 2**).

## 6 Role of lactate in suppression of immunity

The immune system protects the body from damage caused by cancer or other pathogens by detecting and eliminating their respective cells (41). A special category of cells, including macrophages, natural killer cells (NK), and cytotoxic T lymphocytes, identifies and eliminates foreign cells (64). However, recent clinical studies have reported that immune cells fail to recognise tumour cells (65). Malignant cells in the TME remodel adaptive and innate immune responses for their own benefit. Various factors in the TME acts on the immune cells to remodel their metabolic, genetic, and epigenetic level and slowly transforms into resistant to immune cells (66). Owing to the high metabolic rate inside the TME, tumour cells secrete metabolic products such as lactate which favour cancer cells by suppressing immunity.

Studies have also reported that immunogenic cells in the TME assist the tumor cells in various ways to promote growth and development. In their comprehensive review, Wang described lactate as the main onco-metabolite that causes inactivation of immune cells and, thus, suppression of immunity. They reported that lactate acidosis in the TME suppressed the polarisation of M1 macrophages into M2 through an epigenetic mechanism. Lactate enters through the MCT-1 transporter and further binds with DNA, causing histone lysine lactylation (Kla) sites and consequently M2 polarisation. Macrophage polarization are very long debated program in context with host immune response in TME. Furthermore, they showed that a high lactate concentration in the TME disrupts the proton gradient in CD8+T cells, hindering the proliferation of effector T cells. Finally, they explained that lactic acid acts as a signalling molecule in both malignant and dendritic cells. Binding of lactate to GPR81 (G protein coupled receptor) present on dendritic cells causes decreased production of cAMP, IL6, and Il12, and suppression of the antigen presentation mechanism. While lactate binds to GPR81 present in cancer cells, it induces drug resistance and augments the expression of programmed death ligand(PD-L1) (67, 68).

Unlike macrophages, neutrophils function as a double-edged sword for cancer cells. On one hand, they induce apoptosis by employing hydrogen peroxide (H_2_ O_2_), reactive oxygen species (ROS), and tumour necrosis factor-α (TNF-α). On the other hand, these cells secrete inflammatory mediators, initiate angiogenesis, and thus promote tumour growth and development. Neutrophils can assist tumour cells in immune surveillance through extracellular trap formation (69). Hypoxia-induced lactate acidosis helps mobilise neutrophils towards the TME. Khati-Massalha et al., proposed a model for describing the mobilisation of neutrophils from the bone marrow in response to lipopolysaccharide (LPS) lactate in a mouse model of inflammation. They observed that neutrophil mobilisation significantly increased in the bone marrow in response to LPS injection. LPS acts on the toll-like receptors present on neutrophils which stimulates glycolysis and enhances lactate production and secretion through the MCT-4 receptor. Secreted lactate binds to the GPR81 receptor on endothelial cells and decreases surface VE-cadherin levels, leading to higher bone marrow endothelial cell permeability in neutrophils (70). The same mechanism must be used by lactate in hypoxic tumours to enhance the permeability and infiltration of neutrophils in the TME.

Recently, Deng and colleagues, in their study on hepatocellular carcinoma cells, reported that neutrophils in the TME of solid tumours suppress T cell cytotoxicity. Lactate in the TME enters neutrophils *via* the MCT-1 transporter and enhances the expression of the death ligand PD-L1 *via* the activated NFkβ/COX-2 pathway. The study also reported that PD-L1 expression in neutrophils can be reduced by the selective cyclooxygenase-2(COX-2) inhibitor celecoxib. Furthermore, the combination of celecoxib and lenvatinib potentiates its anti-angiogenic action in hepatocellular carcinoma (71). However, it could be beneficial to minimise the level of PD-L1 expressing neutrophils in the TME. To evaluate the effect of PD-L1 inhibition, Guen et al. induced urothelial carcinoma in experimental animals by injecting MB49 murine urothelial cancer cell lines, and evaluated the tumour morphology with haematoxylin and eosin staining and immunohistochemistry. Animals were treated with PD-L1 antibody alone and in combination with 1-palitoyl-2-linoleoyl-3-acetyl-rac-glycerol (PLAG). The results of this study showed that the number of neutrophils infiltrating the tumour tissue decreased considerably in the PLAG- and PD-L1 antibody-treated groups and the population of cytotoxic T-cell cells increased substantially (72). This study clearly validated the role of PD-L1 in immune suppression and its association with lactate acidosis in solid hypoxic tumours. We can assume the same conditions in the hypoxic cancer of mammary gland as excessive accumulation of lactate is also reported in the TME of mammary gland tumors.

Gottfried et al. previously reported that lactic acid causes further differentiation of monocytes into tumour-associated dendritic cells (TADCs). They generated multicellular tumour spheroids (MCTS) using various tumour cell lines and cultured monocytes in the presence of granulocyte macrophage colony-stimulating factor (GM-CSF) and IL-4. They observed that monocytes penetrated MCTS and differentiated into TADCs. They concluded that external sources could modulate monocyte phenotype. Furthermore, melanoma cells and prostate cancer MCTSs were co-cultured with lactic acid. They observed that the phenotype of DCs is similar to that of TADCs (73).

The adaptive immune system CD8+T cells (cytotoxic T cells) also become inactive in the acidic medium of hypoxic tumours. Fischer et al. collected serum samples from patients with malignant cancers such as breast cancer, gastric cancer, and lung cancer. Serum lactate levels were measured in individual samples and correlated with cytotoxic T-cell activity. The results of this analysis showed that T cell activity and the production of cytokines, such as interferon- γ (IF-γ)), decreased significantly in the acidic TME. T cells rely on glycolysis, and the resultant lactate is exported through the MCT-1 transporter; however, lactate acidosis imparted by hypoxic tumours causes inhibition of MCT-1 and thus accretion of lactic acid in T cells, and consequently their inactivation (74). Lactate also cause suppression of CD8+ T cell indirectly by inducing expression of TGF-β. Gu et al. reported that lactate in TME regulate the expression of TGF-β which helps the in suppression of immunity through T-regulatory cells (75). Further, Gunderson et al. illustrated the role of TGF-β in suppressing the activation of CD8+T cells through CXCR3. In their experiment on transgenic mice and tumour cell lines, they observed that TGB-β receptor-knockout mice exhibited higher CXCR3 and CD8+ T cell tumour infiltration than TGF-β receptor-positive cells. It was concluded that concluded that acidosis induced TGF expression which suppressed the CXCR3 expression and thus activation of CD8+ T cells in TME (76). These findings clearly showed that lactate can suppress the CD8+ T cells.

Natural Killer (NK) cells also destroy damaged, infected, and cancerous cells. This action is executed by NK receptors. All cells without major histocompatibility class-1 (MHC-1) on their surfaces were recognised and killed. NK cells release cytoplasmic granules containing granzyme and perforin which induce cell lysis in foreign cells (77, 78). Numerous studies have reported that cancer cells show decreased levels of MHC-1 which results in inactivation of NK cells (79). Lactate leads to the apoptosis of NK cells by decreasing intracellular pH which results in mitochondrial dysfunction and consequently programmed cell death. Lactate also blocks the synthesis and secretion of IFN-γ and interleukin-1 (IL-1) by NKT cells in the TME (41).

Husaain et al., studied the effects of lactate on MDSCs and NK cell function in a syngeneic Pan02 murine pancreatic cancer model. Considering that LDH-A is responsible for the conversion of glucose into lactate, LDH-A-deficient Pan02 (pancreatic cell lines) cancer cells were prepared by a knockdown process using Lentiviral vector –mediated hairpin RNA and injected into C57BL/6 mice. They observed smaller tumor in C57/6 mice compared than Pan02 treated mice. In addition, they noted a decrease in the number of MDSCs and NK cells in the spleens of LDH-A gene-deficient mice. *In vitro* exogenous supplementation with lactate increased the frequency of MDSC generation from mouse bone marrow, along with GM-CSF and interleukin (IL-6). *In Vitro* pre-treatment of NK cells with lactate impedes their cytotoxicity in both humans and mice. The results also showed a reduction in the expression of perforin, granzymes, and NKp46 proteins in the NK cells. Furthermore, they noted that mice developed fewer tumours when given access to only a ketogenic diet. This study clearly demonstrated the immunosuppressive role of lactate in MDSCs and NK cells (80). Serganova et al. first reported that LDH-A inhibition modulates the tumour immune response. They also noted reduced expression of HIF-1, Hexokinase 1and 2, and VEGF in LDH-A knockdown mice. Again, this study established a clear relationship between hypoxia, LDH-A, and tumour immunity (81).

## 7 Role of lactate in development of resistance against chemotherapy and radiotherapy

Lactate is currently recognised as the major oncometabolite which helps cancer cells to develop resistance to chemotherapy. Various studies have reported the acquired resistance caused by lactate. Lactate acidosis in TME can activate various receptors that contribute to the development of resistance to chemotherapy. Although c-MET is a receptor tyrosine kinase (RTK) that plays a vital role in the proper growth and development of normal cells, aberrant activation can sustain tumour growth and metabolism (82). Recently, Apicella et al. were the first to report that lactate in the TME assists cancer cells in acquiring and developing resistance to targeted therapies. According to the author, continuous and long-term treatment with Met or EGFR tyrosine kinase inhibitors caused excessive production of lactate which is taken up by CAF through the MCT4 transporter. Lactate in CAF activates NF-κB which enhances the transcription of hepatocyte growth factor (HGF). Previous studies have reported that HGF prevents apoptosis in both normal and cancer cells induced by various stimuli (83). When secreted into the TME, HGF binds to the MET receptor on the cancer cells and overcomes the inhibitory effect of receptor tyrosine kinase inhibitors (TKIs), such as sunitinib (84).

Another study conducted by Govon et al. demonstrated and noticed the resistance-imparting behaviour of lactate to cisplatin therapy in cancer cell lines. They selected a cell line with the potential to grow in lactate-containing culture medium and simultaneously treated it with cisplatin. It is well known that cisplatin is a platinum compound having the DNA damaging potential in cancer cells. The results of this study showed that cells grown in the lactate medium had a very low impact on cisplatin therapy. The efficacy of cisplatin was significantly reduced in the lactate-supplemented cells. Moreover, cells treated with lactate exhibited reduced DNA damage and increased levels of DNA repair genes. They reported that lactate can impart resistance to chemotherapeutic agents (85).

Park et al., studied the effects of lactate on breast cancer cell lines and reported that some cancer cells can stop using glucose and begin utilising lactate as an energy substrate, which confers resistance to PI3K/mTOR inhibitors. Furthermore, they reported that oestrogen-related receptor alpha (ERR-α) regulates the expression of various genes involved in lactate utilisation and uptake. Notably, the efficacy of inhibitors of the PI3K/mTOR pathway was substantially increased both *in vitro* and *in vivo* when ERRα antagonists were used (86).

In another study conducted by Qi Dong et al., etoposide administration exacerbated ROS production which indirectly reprogrammed glucose metabolism and enhanced lactate synthesis in NSC-LCs. The resultant lactate acidosis confers resistance to cancer cells by enhancing the upregulation of multiple resistance-associated protein 1(MRP-1), an AT-binding cassette (ABC) transporter protein. MRP-1 acts as a drug efflux pump in cancer cells and its expression increases in many types under the guidance of lactate acidosis (87).

Qu et al. reported in their study that lactate enhanced resistance to oxaliplatin in colorectal carcinoma (CRC) patients co-infected with *Candida tropicalis (C. tropicalis)*. They induced CRC carcinoma *via* xenografting of the colorectal cancer cell line SW480 in mice and validated their findings using various parameters such as apoptosis, immunohistochemistry, and western blotting. The results of this study showed a substantial increase in tumour burden in experimental animals treated with oxaliplatin and infected with *C. tropicalis*. Furthermore, they reported that lactate significantly altered the expression of mismatch repair proteins(MMR) as MSH1 and MSH2 through activation of the GPR81-cAMP-PKA-CREB axis (88). Leslie Amaral et al. worked in the same direction and also reported that lactate impart resistance to chemotherapeutic agents. They cultured *Saccharomyces cerevisiae* (S. cerevisiae) BY4741 cell lines in the presence and absence of glucose and lactate, and treated them with cisplatin. After 180 min of exposure to the respective treatments, they observed a reduced sensitivity to cisplatin in the presence of lactate. Furthermore, western blotting results showed higher phosphorylation of Rad4p in lactate medium-cultured cells, although no change in histone acetylation was observed (89). It is previously reported that Rad4p (a DNA repairing protein that belongs to xeroderma pigmentosum family) has important role in nucleotide excision repair (90). To understand the effect of lactate on gene expression in cancer cells, Govoni et al. selected a cancer cell line capable of growing under glucose-deprived conditions, and evaluated the effect of lactate acidosis on the DNA-damaging potential of cisplatin. They observed that the low efficacy of cisplatin in lactate-exposed cells was due to enhanced DNA recombination and the upregulation of DNA repair genes. They identified various genes in lactate-treated cell lines that participate in the mismatch repair and nucleotide excision pathways and restore cisplatin-induced DNA damage (85).

Xiaoping et al. collected samples from patients with NSCLC undergoing cisplatin treatment and analysed them using immunohistochemistry and RT-qPCR. The results showed that higher expression of osteopontin (OPN) protein which directly contributes to LDHA expression. Higher LDHA expression further enhances lactate acidosis, imparting resistance to cisplatin therapy. The same correlation between, lactate, and resistance to cisplatin was confirmed through *in vitro* studies conducted on SK-MES-1 and A549 cell lines (91). This study established a new relationship between OPN and lactate acidosis and resistance. OPN plays a crucial role in malignancy (92).

**Figure 3:**
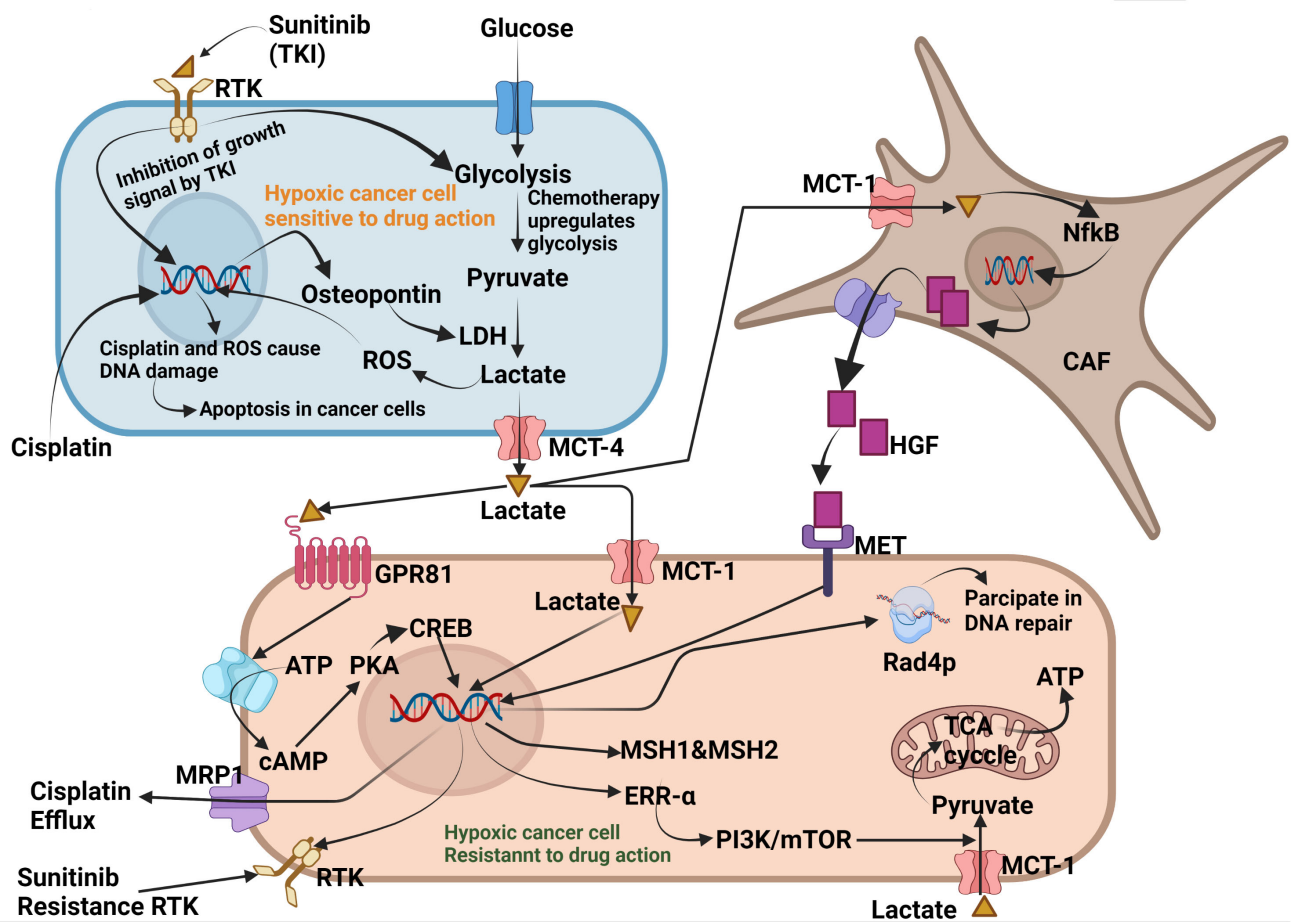
Molecular mechanism exploited by Lactate for developing resistance against chemotherapy. Exposure to cisplatin and tyrosine kinase inhibitors enhances cytoplasmic levels of osteopontin in cancer cells. Osteopontin enhances LDH expression which enhances glucose fermentation into lactate. Lactate from sensitive cancer cells is secreted into the TME *via* MCT4, where it is taken up by neighbouring cancer cells and cancer-associated fibroblasts (CAF). In lactate, NF-κB which enters the DNA, enhances the synthesis and secretion of the cytokine HGF. HGF from the TME binds to the MET receptor present on neighbouring cancer cells, making the receptor insensitive to tyrosine kinase inhibitors such as sunitinib. Lactate can also bind to GPR81 which enhances the expression of DNA mismatch repair proteins, such as MSH1 and MSH2, through the GPR81-cAMP-PKA-CREB axis. Cisplatin enhances cytoplasmic levels of oestrogen response receptor-α(ERRα), which helps cancer cells utilise lactate as an energy substrate. Lactate also phosphorylates Rad4p, which participates in the repair of cisplatin-induced DNA damage and ROS. Created by Biorender.

These studies clearly demonstrated that lactate plays a pivotal role in the emergence of resistance in cancer cells. It exploits various molecular pathways to induce resistance in cancer cells. When cancer cells are treated with chemotherapeutic agents such as cisplatin or tyrosine kinase inhibitors, they initially respond well, suggesting that they are drug-sensitive. However, continuous torture by chemotherapeutic agents forces cancer cells to develop a mechanism that counteracts drug response. Drug-sensitive cancer cells show increased cytoplasmic levels of OPN, which upregulates LDH expression. Overexpression of LDH enhances glucose oxidation to lactate which is pumped into the TME by MCT4. Lactate from the TME is absorbed by neighbouring cancer cells and CAF *via* MCT1. In CAF, lactate enhances NF-κB which acts on DNA, upregulates the synthesis and secretion of HGF cytokine which acts on the MET receptor and makes the tyrosine kinase receptor insensitive to tyrosine kinase inhibitors such as sunitinib and makes the cancer cells resistant to TKIs. Lactate from the TME enters directly through GPR81 and enhances the expression of mismatch repair genes such as MSH1 and MSH2 through the GPR81-cAMP-PKA-CREB axis. Lactate entering MCT1 acts on DNA and enhances the expression of multiple resistance transporter-I (MRT1) which acts as an efflux pump for cisplatin. Cisplatin exposure enhances ERRα expression, which allows cancer cells to oxidise lactate in the TCA cycle as an alternative to glucose. Lactate enhances the phosphorylation of Rad4P, which helps to repair DNA damage caused by cisplatin or ROS. Rad4P belongs to xeroderma pigmentosum group-C family-participate in nucleotide excision repair (**Figure 3**). Phosphorylated Rad4P carry out the repairing of damaged DNA of cancer cells treated with chemotherapeutic agents like cisplatin. This is how lactate acidosis renders the cancer cells insensitive to chemotherapeutic agents. Physician can enhance the efficacy of chemotherapeutic by co-administration of inhibitors of HGF and Rad4P.

## 8 Lipid reprogramming of cancer cells

### 8.1 Hypoxia induce lipid droplet accumulation in cancer cells

Recent work has pointed out that lactate is the principal oncometabolite which is essential for the continuous running of glycolysis and oxidative phosphorylation and as a precursor for biosynthetic processes in cancer cells (93). Lactate has also been reported to play an important role in gene regulation and expression, partitioning of energy substrates, and the regulation of cellular redox homeostasis. Although much has been reported regarding the role of lactate in the metabolic reprogramming of glycolysis, drug resistance, immune suppression, and invasiveness, very little attention has been paid to its role in lipid reprogramming. Recent studies have reported that lactate reprograms lipid metabolism in cancer cells (94). As lipids are indispensable for rapidly dividing cancer cells to form their plasma membrane along with other cell organelles (95). Fatty acids are abundantly required for nascent cancer cell membrane which cannot be accomplished alone by *de novo* fatty acid synthetic pathway (96). Cancer cells utilise lactate to produce more fatty acids. In addition, various studies also have reported that hypoxia in solid tumour induces the formation of lipid droplets which are can be used for energy production during oxygen availability (97). However, exact the mechanisms by which cancer cells use lacate in fatty acid synthesis remains unexplored.

However, recent studies have documented a role for lactate in lipid reprogramming. Corbet et al. reported that tumour acidosis in cancer cells favours lipid oxidation and synthesis under lactate acidosis. The results of the study also documented that acetyl-co-A derived through β-oxidation of fatty acids not only fuels the Krebs cycle but it also checks ROS production in mitochondria. In addition, acetyl-co-A resulting from β-oxidation lead non-enzymatic hyperacetylation of mitochondrial complex-I. Overall, it was postulated that fatty acid oxidation and synthesis takes place concomitantly in cancer cells in acidic environment which was enabled by the sirtuin-mediated deacetylation of histones and consecutively downregulation of acetyl-CoA carboxylase 2(ACC2) (98). Pierre Sonveaux reported in their study that TME is heterogeneous with respect to oxygen and nutrient availability. The cells near the blood vessels have adequate nutrient and oxygen availability, whereas those located in the middle and periphery of the tumour are deficient in oxygen; hence, they are called hypoxic tumour cells. Hypoxic cancer cells metabolise glucose only through glycolysis, resulting in the accumulation of excess lactic acid, which is pumped into the extracellular environment, making it more acidic. Generally, normal cells undergo apoptosis in the presence of oxygen and nutrient deficiency. Cancer cells are immortal and do not initiate programmed cell death but develop a metabolic symbiosis with normoxic cancer cells (OXOPHOS cells). Lactate generated by hypoxic malignant cells is utilised by OXOPHOS cells and is further used in the TCA cycle. Simultaneously, OXOPHOS stops using glucose (Warburg effect) which is retained in hypoxic cancer cells (99). Another study reported the roles of low-density lipoprotein (LDL) and very-low-density lipoprotein (VLDL), but not lactate, in breast cancer progression. When MCF7 and MDB-MB-231 cells were supplemented with LDL and VLDL, angiogenesis in the breast cancer cells was initiated. Although this study did not show any link between lactate and fatty acid synthesis, it proved that rapidly dividing cancer cells can take up circulating lipoproteins to accomplish their fatty acid needs, regardless of the raw material used in the synthesis of these fatty acids (100). Shen et al. reported a role for HIF-1α in LDL and VLDL receptor (VLDLR) regulation. In their study on MCF7, HepG2, and HeLa cells, LDL and VLDL levels were upregulated when cultured under hypoxia. The gene and mRNA expression of VLDLR increased significantly compared with that in cells cultured under normoxia. Furthermore, they confirmed the presence of an operational hypoxia response element (HRE) gene adjacent to the VLDLR gene over +405 exon 1 using dual luciferase and chromatin immunoprecipitation assays. The HRE of VLDLR responds to HIF-1α. In addition, knockdown of HIF-1αand VLDLR genes attenuated lipid accumulation in cultured cells, indicating a direct link between HIF-1α and LDL and VLDL (101). Sundelin et al. observed that VLDLR overexpression in human and mouse cardiomyocytes under hypoxic conditions results in detrimental lipid accumulation. After thoroughly mapping the 50-flanking region on the VLDLR promoter gene, they stated that the hypoxia-mediated increase in VLDLR protein expression depended on the HRE present between 162 to 158bp translation site. This study rejected the previously described hypothesis that PPARc and SP1 binding sites on the VLDLR promoter region are not involved in the hypoxia-induced regulation of VLDLR expression (102).

Previously, Kodndo et al., found that both hypoxia and nutrient scarcity play critical roles in malignancy. However, the role of hypoxia-induced extracellular pH (pHe) in lipid biosynthesis has not been fully elucidated. His study on pancreatic cell lines (PANC-1 and ASPC-1) clearly documented the role of tumour acidosis in lipid biosynthesis. Reduced pHe in the external environment activates sterol regulatory element binding protein-2 (SREBP2) which is released from the endoplasmic reticulum and binds to the sterol response element in the nucleus. This leads to the activation of various genes involved in fatty acid synthesis (103).

In the above text, we have already discussed the study conducted by Corbet et al., who reported the effect of tumour acidosis on cancer progression by enhancing lipid droplet formation. They found that acidic pH encourages autocrine TGF-β signalling, which indirectly enhances the formation of lipid droplets (LD) and further helps in developing anoikic resistance, thus participating in EMT progression. In addition, TGF-β2 activation promotes epithelial-to-mesenchymal transition and lipid metabolism. Furthermore, it stimulated PKC-zeta-mediated translocation of CD36 which further enhanced fatty acid uptake, either stored in the form of triglycerides in the LD or utilised to generate ATP by oxidation. The study also described that distant metastasis can be prevented by inhibiting the mobilisation of fatty acids from the LD (55).

Bensad et al., reported that hypoxia in cancer cells activates various genes which directly and indirectly enhance lipid synthesis and storage. Further results of the study reported that HIF-1α induces the accretion of LDs in hypoxic cancer cells. These LD are rich in triglycerides (TGs) which are degraded to release fatty acids and used in ATP production by β-oxidation in the mitochondria, and can also be used to build the cell membrane of rapidly proliferating cancer cells. The link between LDs formation and hypoxia was established based on the overexpression of several surface proteins located on the LDs membranes. Hypoxia-inducible protein2(HIG2), Adipose differentiation-related protein(ADRP), and Perilipin-3(TIP47) constitute the surface of LDs. Of these, HIG2 and ADRRP are mainly induced by hypoxia. Role of FABP2/3 and ADRP in selective uptake of long-chain fatty acids was observed to be increased in hypoxic cancer cells (97).

Based on the above results, we hypothesised that hypoxia in cancer cells shifts glucose metabolism only through glycolysis. As a result, excess lactate accumulates in cancer cells and is secreted into the extracellular TME. An increase in the proton concentration in the TME stimulates the uptake of protons by nearby cancer cells, resulting in increased acidosis in these cells. Reduced acidosis further stimulates SREBP-1c present in the endoplasmic reticulum and translocates to the nucleus, where it activates genes that are indispensable for fatty acid synthesis. It has also been shown that excess glucose supplements in cancer cells can induce the accumulation of lipid droplets (LD) in cancer cells. Tirinato et al., while working on normal and colorectal cancer stem cells (CR-CRC), found that excess glucose induced LD and ROS production in cancer cells. Excess glucose is converted into palmitate which is further converted into triglycerides and cholesterol and finally encapsulated into LDs. TG and cholesterol from LD have been used for energy production (104).

Fabienne et al. obtained tumour specimens from patients with pancreatic cancer to understand the role of lactate and the expression of the low-density lipoprotein receptor (LDLR). They observed that LDLR expression increased many times in patients with pancreatic cancer. These results further supported that the higher expression of LDLR illustrates that malignant cells are metabolically active and eager to synthesise cholesterol. This further increased the risk of relapse. This study not only reported the role of cholesterol in cancer progression but also suggested that the synthesis and design of novel therapeutic agents against LDLR receptor-inactivating enzymes could be a new approach for the treatment and management of pancreatic carcinoma (105). Studies have also reported a role for cholesterol in cancer progression.

Later, many researchers worked on it to prove this hypothesis. Singh et al. induced breast carcinoma with MNU and DMBA (7-12 Benzanthracene), and a the serum metabolomic profile of experimental animals was analysed using Nuclear magnetic resonance (NMR). A large perturbation in serum metabolites was observed in toxicant treated animals. Overall, conclusion of the study was that hypoxic cancer cells reprogrammed their glycolytic pathway to enhance lactic acid production (106, 107). Interestingly, higher levels of lipids (polyunsaturated fatty acids) were also observed in the same animals with increased lactate level. Enhanced expression of HIF-1α, SREBP-1c, and fatty acid synthase (FASN) was noted with an increase in lactate acidosis (106). Recently, Minami et al. reported that glucose-deprived glioma stem cells (GSC) utilise lactate in the Krebs cycle to generate GTP. It has also been reported that choline, phospholipid, and fatty acid synthesis increases many-fold upon lactate supplementation in glucose-deprived cancer cells. In addition, cancer cells exhibit increased aggressiveness and metastasis following lactate consumption (108). This study clearly shows that cancer cells can reuse lactate for fatty acid synthesis. Luci et al. observed the same phenomenon in glial and neuronal cells. Under stress conditions, glial cells utilise glucose which is converted to lactate which is pumped out extracellularly and taken up by the neuron cells through the MCTs transporters and metabolised back to pyruvate-citrate so that it can be used in fatty acid synthesis. Excess fatty acids are not utilised by these cells, but are exported through apolipoproteins (ApoE/D) and stored in the form of LDs after being converted into TG and cholesterol. Under stressed conditions, glial cells and neuronal cells develop a metabolic symbiosis to minimise the ROS effect because excessive ROS can lead to neuronal degeneration in CNS disorders, such as Alzheimer’s disease (109).

### 8.2 Role of lactate in causing lipid reprogramming in cancer cells

To understand lipid reprogramming in cancer cells, we must sit in the tumour microenvironment to closely observe metabolic changes at the molecular level in solid tumours. Furthermore, the TME is different in larger tumours than in smaller ones. First, we will understand the tumour microenvironment of smaller tumours. When a cell transforms from normal to malignant, it is located near the blood vessel, and all the tumour cells arising from these cancer cells receive adequate amounts of nutrients and oxygen. Although all cancer cells in the tumour milieu are capable of metabolising glucose by glycolysis and OXPHOS, they prefer to metabolise glucose by glycolysis (Warburg effect). Consequently, excess lactate accumulates in tumour cells, which is dumped outside the TME through MCT-4), from where it is immediately washed away by the circulating fluid. This metabolic plasticity, which is maintained by cancer cells, is necessary for building the biomass. Rapid division of cancer cells requires replication, which requires ribose sugar as the major metabolite. Because ribose and other sugars are only formed by the pentose phosphate pathway (PPP), glycolysis is mandatory to run PPP continuously. The second most important requirement is for fatty acids. Fast running glycolysis yields extra pyruvate which in part is converted into acetyl-CoA and converted into citrate which is further utilised in fatty acid synthesis. Thus, palmitic acid is further converted into monounsaturated fatty acids (MUFA) and polyunsaturated fatty acids (PUFA). These are further altered to form membrane phospholipids, such as phosphatidylcholine, phosphatidylinositol, and sphingolipids, which are used in the construction of plasma membranes of proliferating cancer cells. This is how glycolysis is reprogrammed to enhance lipid synthesis in cancer cells when the tumours are small.

Now, consider the TME of a solid hypoxic tumour. Unlike smaller tumours, the microenvironment in larger tumours is different in that some cancer cells are located near blood vessels to obtain sufficient amounts of nutrients and oxygen; hence, they are called OXPHOS cells. Some cells are located in a distant area at the periphery of the tumour and receive sufficient nutrients or oxygen; hence, they are called hypoxic cancer cells. In addition, there is a third category of cells located between OXPHOS and hypoxic cancer cells, which receive a partial amount of nutrients and oxygen, and are hence called moderately hypoxic cancer cells. Hypoxic cancer cells develop metabolic symbiosis to sustain nutrient- and oxygen-deficient environments. OXPHOS cells do not use glucose, unlike OXPHOS cells in small tumours, but they retard glycolysis. Spared glucose is then transported to severely hypoxic cancer cells. Severely hypoxic cancer cells can only metabolise glucose by glycolysis and thus produce excess lactic acid which is immediately exported to the TME, reducing its pH from 7.4 to 6.8. Continuously exported lactate flows towards OXPHOS cells and is taken up by these cells through the MCT-1 transporter. OXPHOS cells convert lactate back into pyruvate which is then utilised in the Krebs cycle. This phenomenon is known as the reverse Warburg effect (RWE). Lactate-derived pyruvate is further utilised for ATP production by the electron transport system. This also fuels the fatty acid synthesis pathway. The excess citrate produced is then converted into palmitate which is further modified into complex lipids such as triglycerides and cholesterol. Cholesterol is further utilised in the biosynthesis of signalling molecules, hormones, and cell membrane lipids such as phosphatidylinositol, phosphocholine, and sphingolipids. Fatty acids converted into LDL/VLDL are transported towards the hypoxic cancer cells and accumulated in the form of LDs (110).

**Figure 4:**
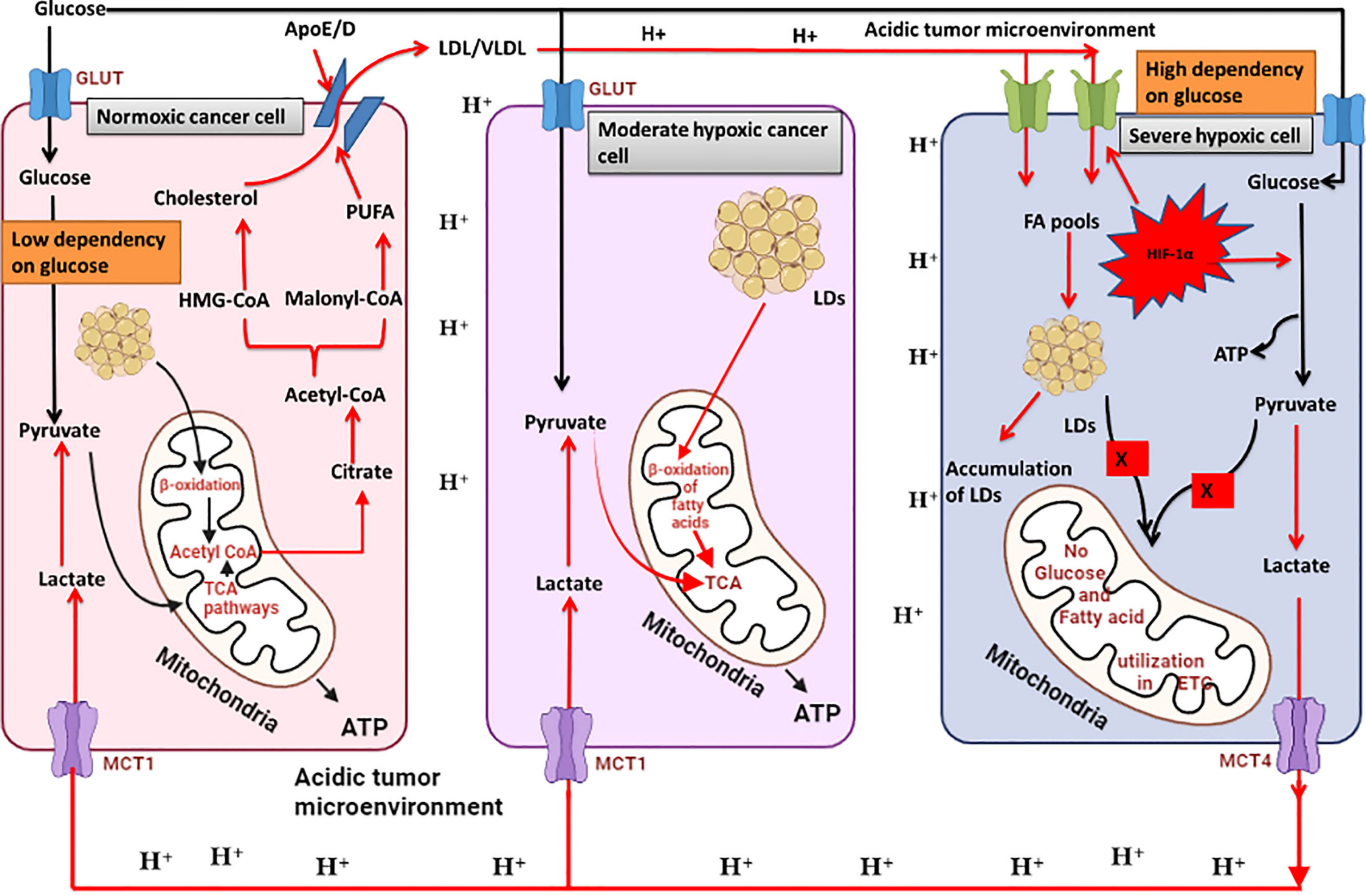
Metabolic symbiosis in cancer cells to utilize glucose and lactate for fatty acid synthesis and utilisation. Severely hypoxic cancer cells can oxidise glucose only through glycolysis which results in excessive production of lactate secreted in the tumour microenvironment. Enhanced glycolysis in these cells also results in lipid droplet accumulation. OXPHOS cancer cells can use lactate for oxidative phosphorylation by converting it back to pyruvate. Therefore, OXPHOS cells and severely hypoxic cancer cells develop a metabolic symbiosis for glucose and lactate utilisation. Excess lactate in OXPHOS cells is released as citrate which is utilised in fatty acid synthesis. Moderately hypoxic cancer cells can utilise fatty acids for ATP production through β-oxidation of fatty acids. Fatty acids accumulate in severely hypoxic cancer cells and are transported towards OXPHOS cells, where they are partly converted into fatty acids and utilised in β-oxidation for energy production. A large portion is converted into polyunsaturated fatty acids (PUFAs) which are further incorporated into plasma membrane synthesis. Created by Biorender.

From the above discussion we can conclude that cancer cells in TME develop a metabolic symbiosis for synthesis and utilization of fatty acids. Severely hypoxic cancer cells can extract energy only from glycolysis (high dependency on glucose), hence produce and secrete excess of lactate in the TME. Lactate from TME flows towards the normoxic cancer cells (low dependency on glucose) and is utilized in the TCA cycle after being converted back to pyruvate. Lactate derived citrate is also utilized in *de novo* fatty acid synthesis. In these cells, simple fatty acids are also converted into complex polyunsaturated fatty acids (PUFAs). PUFAs and Triglycerides are further converted into cholesterol and other structural lipids. Since severely hypoxic cancer cells cannot make complex lipids, these cells take up the complex lipids prepared by the normoxic cancer cells. Continuous accumulation if lipids in the hypoxic cancer cells are stored in the form of LDs. Hypoxic cancer cells can utilize FAs stored in the LDs during hypoxia re-oxygenation in β-oxidation to meet the high energy demand during rapid growth phase (**Figure 4**).

## 9 Conclusions

Cancer cells struggle with nutrients and oxygen as the tumour size increases to a reasonable size (3). Lactate secreted by hypoxic cancer cells initiates angiogenesis, and severely hypoxic cancer cells enhance the synthesis and secretion of VEGF which acts on epithelial cells present in nearby blood vessels. Binding of VEGF to ECs leads to their differentiation into stalk ECs and tip ECs (111). Lactate from the TME is also taken up by TAM, where it activates HIF-1 which enters the nucleus of TAM and activates various genes involved in angiogenesis. TAM secrete VEGF and TNF-α which bind to the tip of EC cells and enhance their proliferation. MMP secreted by TAM acts as a tissue-digesting enzyme, creating space for vessel formation (112). Lactate secreted from hypoxic cancer cells also enters stalk ECs, where it enhances the expression of Gas6, VEGF, Ang-1, and IL-8, which act on their respective receptors present on stalk cells in an autocrine mechanism, activate Myc, cyclin-D1 genes and subsequently induce cell division in stalk ECs. Tip ECs also secrete the Dll4 ligand which further enhances the proliferation of stalk ECs through the Notch signalling pathway. Lactate-mediated angiogenesis in solid tumours can be blocked by designing VEGF, Dll4, Gas6, IL-8, and Ang-1 inhibitors (113).

Tumour cells utilise lactate to initiate EMT. Lactate enhances TGF-β2 expression, which activates CAD and VIM gene expression. The inhibitors of TGF-β2, CAD, and VIM can prevent invasiveness. EMT can be further blocked by inhibiting LCFA uptake using transporter blockers (95, 114).

To protect themselves from chemotherapeutic agents, cancer cells develop resistance by using lactate as a mediator. Resistance to lactate can be abrogated by designing therapies against HGF cytokines which enhances HGF cytokines. MRT-1 inhibitors can prevent drug efflux from the target cells, rendering them sensitive to chemotherapy. Drugs that prevent Rad4p phosphorylation and antagonise MSH1 and MSH2 can overcome drug resistance by interfering with DNA repair mechanisms. ERR-α antagonists halt lactate utilisation in the mitochondria for energy production.

Cancer cells are self-sufficient to fulfil their fatty acid requirements. The metabolic symbiosis between OXPHOS, moderately hypoxic, and severely hypoxic cancer cells helps foster a harsh environment. Glucose is utilised by severely hypoxic cancer cells and rapidly fermented into lactate. Lactate is pumped into the tumour milieu and enters OXPHOS and moderately hypoxic cancer cells. Lactate is utilised by OXPHOS and moderately hypoxic cancer cells to fuel fatty acid synthesis and TCA cycle. Fatty acids formed by hypoxic and moderately hypoxic cancer cells are further modified for membrane lipid synthesis. Fatty acids can also be used in angiogenesis and invasion. Inhibitors of fatty acid synthesis are also beneficial in cancer therapy. Fatty acid synthesis inhibitors like HMG-CoA inhibitors (Statins), can be repurposed along with other anti-cancer drugs.

## Author contributions

LS and LN, performed the major writing work, and designed the graphical illustration mechanism. SM, SR, AD, and MA proofread the manuscript. MS and MC conceived the idea, performed final proofreading and prepared the final manuscript. All authors contributed to the article and approved the submitted version.

## Glossary

**Table:**

TME	Tumour microenvironment
HIF-1α	Hypoxia-induced factor-1α
TCA	Tricarboxylic acid
NSC-LC	Non-Small Cell Lung Carcinoma
CD8+ T cells	cytotoxic T cells
NK	Natural Killer cells
DC	Dendritic cells
NF-kB	Nuclear factor kappa-B
Cln-1	Claudin-1
LDHA	Lactate dehydrogenase
MNU	N-Methyl-N-Nitrosourea
NMR	Nuclear magnetic resonance
VEGF	Vascular endothelial growth factor
PGA-1	Plasminogen activator inhibitor-1
PDGF-B	Platelet-derived growth factor-B
FLT-1 and FLK-1	Fms Related Receptor Tyrosine Kinases
MMP-2	Matrix metalloproteinases
Tie2	A receptor tyrosine kinase
ANG-1 and ANG-2)	Angiopoietins
EMT	Epithelial to mesenchymal transition
FGF-2	Fibroblast growth factor-2
PIGF	Placental growth factor
BAECs	Bovine aorta endothelial cells
MCT-1 and 4	Monocarboxylate transporter-I &4
TAM	tumour-associated macrophages
HUVECs	cultured human umbilical endothelial cells
Axl	A tyrosine kinase receptor
DLL4	Delta-like Ligand-4
EC	endothelial cells
GPCR	G-protein coupled receptor
Arg-1	Arginase-1
ICER	Inducible cAMP early repressor
Fizz-1	Resistin-like molecule alpha1
LDHB	Lactate dehydrogenase B
OSC	Oral Squamus Cell Carcinoma cells
TGF-β	transforming growth factor-β
ZEB1	Zinc finger E-box-binding homeobox 1
CDH2	Cadherin-2
VIM	Vimentin
LCFA	Long chain fatty acids
CAF	Cancer-associated fibroblasts
ATM	Ataxia-telangiectasia
GLUT-1	Glucose Transporter-1
DSB	double-strand-break
pmCIC	Plasma membrane citrate carrier
MRPL13	Mitochondrial ribosomal protein L13
ETC	Electron transport chain
OXOPHOS	Oxidative phosphorylation
ROS	Reactive oxygen species
ATP	Adenosine triphosphate
CCL4,5,6,10,17	Motif chemokine 4,5,6,10,17
PD-L1	Programmed death ligand
H2H2	Hydrogen peroxide
TNF-α	Tumor necrosis factor-alpha
LPS	Lipopolysaccharide
COX-2	cyclooxygenase-2
PLAG	1-palitoyl-2-linoleoyl-3-acetyl-rac-glycerol
TADCs	Tumour-associated dendritic cells
MCTS	multicellular tumor spheroids
GM-CSF	Granulocyte macrophage colony stimulating factor
	
	
MHC-1	major histocompatibility class-1
c-MET	A receptor tyrosine kinase
HGF	Hepatocyte growth factor
TKIs	Tyrosine kinase inhibitors
ERR-α)	Estrogen-related receptor alpha
MRP-1	Multiple resistance-associated protein 1
ABC	AT-binding cassette
CRC	Colorectal carcinoma
MMR	Mismatch repair proteins
ACC2	Acetyl-CoA carboxylase 2
LDL	Low density lipoprotein
VLDL	Very low density lipoprotein
VLDLR	VLDL receptor
HRE	Hypoxia response element
pHe	Extracellular Ph
SREBP2	Sterol regulatory element binding protein-2
TGs	Triglycerides
LDs	Lipid droplets
CR-CRC)	Colorectal cancer stem cells
LDLR	Low-density lipoprotein receptor
GSC	glioma stem cells
ApoE/D	Apolipoproteins
PPP	pentose phosphate pathway
PUFA	Polyunsaturated fatty acids.
